# Scoliosis Related Information on the Internet in China: Can Patients Benefit from This Information?

**DOI:** 10.1371/journal.pone.0118289

**Published:** 2015-02-17

**Authors:** Hongda Bao, Feng Zhu, Fei Wang, Zhen Liu, Mike H. Bao, Shouyu He, Zezhang Zhu, Yong Qiu

**Affiliations:** 1 Spine Surgery, The Affiliated Drum Tower Hospital of Nanjing University Medical School, Nanjing, China; 2 Geisel School of Medicine at Dartmouth, Hanover, New Hampshire, United States of America; Leibniz Institute for Prevention Research and Epidemiology (BIPS), GERMANY

## Abstract

**Background:**

There has been an increasing popularity of searching health related information online in recent years. Despite that considerable amount of scoliosis patients have shown interest in obtaining scoliosis information through Internet, previous studies have demonstrated poor quality of online information. However, this conclusion may vary depending on region and culture. Since China has a restricted Internet access outside of its borders, the aim of this study is to evaluate the quality of scoliosis information available online using recognized scoring systems and to analyze the Internet as a source of health information in China.

**Methods:**

A survey-based questionnaire was distributed to 280 respondents at outpatient clinics. Information on demographics and Internet use was collected. Binary logistic analysis was performed to identify possible predictors for the use of Internet. In addition, the top 60 scoliosis related websites assessed through 4 search engines were reviewed by a surgeon and the quality of online information was evaluated using DISCERN score and JAMA benchmark.

**Results:**

Use of the Internet as a source for scoliosis related information was confirmed in 87.8% of the respondents. College education, Internet access at home and urban residence were identified as potential predictors for Internet use. However, the quality of online scoliosis related information was poor with an average DISCERN score of 27.9±11.7 and may be misleading for scoliosis patients.

**Conclusion:**

The study outlines the profile of scoliosis patients who use the Internet as a source of health information. It was shown that 87.8% of the scoliosis patients in outpatient clinics have searched for scoliosis related information on Internet. Urban patients, higher education and Internet access at home were identified as potential predictors for Internet search. However, the overall quality of online scoliosis related information was poor and confusing. Physician based websites seemed to contain more reliable information.

## Introduction

The Internet has become a widely used tool for communication and a source of information. Searching for health-related information on the Internet is widespread among patients [1]. In 2010 alone, 52.5% of Internet users searched for information on health topics [2]. In many cases, the information helps patients understand their illness and may be a good source of support if the websites consulted contain the information the patients need [3]. While in other cases, the information online are a concern because they present incorrect information and biased perspectives, making outpatient consultation more challenging [4]. Recent polls reported that among those who use the Internet to assess health-related information, 86% believe the health information to be reliable with 64% reporting that Internet information impacted their medical decision [5,6]. A better understanding of how the Internet is used as a source of health information by scoliosis patients, the factors linked to the use of Internet and the quality of online information will allow for more effective communication between clinicians and patients.

Patients’ demand of online information and their Internet usage vary drastically depending primarily on age, education background and their economic condition [1,7]. With respect to scoliosis, Baker *et al*.[8] reported that a history of corrective surgery and possession of health insurance were two independent positive predictors of Internet usage in patients with scoliosis in Ireland. In addition, Wellburn *et al*.[9] reported that the quality of scoliosis related information on the Internet had been demonstrated to be poor and unregulated. Nason *et al*. [10] demonstrated that the poor quality of online information could contribute to patients’ false interpretation and misled understanding of their personal health.

Recent studies also showed that there are considerable regional and disease-related differences in the use of Internet information [11,12]. The region as either a developing and developed country was not the only factor associated with general Internet access. Other factors, such as cultural differences, the number of accessible websites in local languages and the quality and accessibility of general health services were also important considerations [11]. The previous studies concerning scoliosis related online information were carried out mainly in developed countries [4,8,10,13]. Factors related to the use of Internet in a developing country were not well documented in these studies; specifically, there was limited information on the quality of online scoliosis related information in China. The aim of this study was to evaluate the quality of information available on the Internet using recognized scoring systems and to determine whether the information was useful to patients. Another objective of this study was to analyze the factors related to the use of Internet as a source of health information in China, an example of a developing country.

## Materials and Methods

### Ethics Statement

The study was approved by the ethics committees of the Affiliated Drum Tower Hospital of Nanjing University Medical School. The investigation has been conducted according to the principles expressed in the Declaration of Helsinki. Written informed consents have been obtained from all participants.

### Subjects

The guardians of 280 scoliosis patients who visited our outpatient clinic from March 2013 to August 2013 were recruited into this study. The average age of our patients was 12.86 years of age (range: 3–17 years) and that for the guardians of the patients was 36.72 years of age (range: 25–58 years).

### Analysis of questionnaire

A cross-sectional descriptive study using individual, questionnaire-based interviews was carried out. A questionnaire adapted from a previously used model by Baker et al.[8] was used to collect basic demographic information and information regarding internet use and access (S1 Questionnaire). Participants were asked to complete the questionnaires before the routine care procedure and a 100% response rate was achieved.

### Instrumentations for scoring of scoliosis-related websites

**Fig 1:**
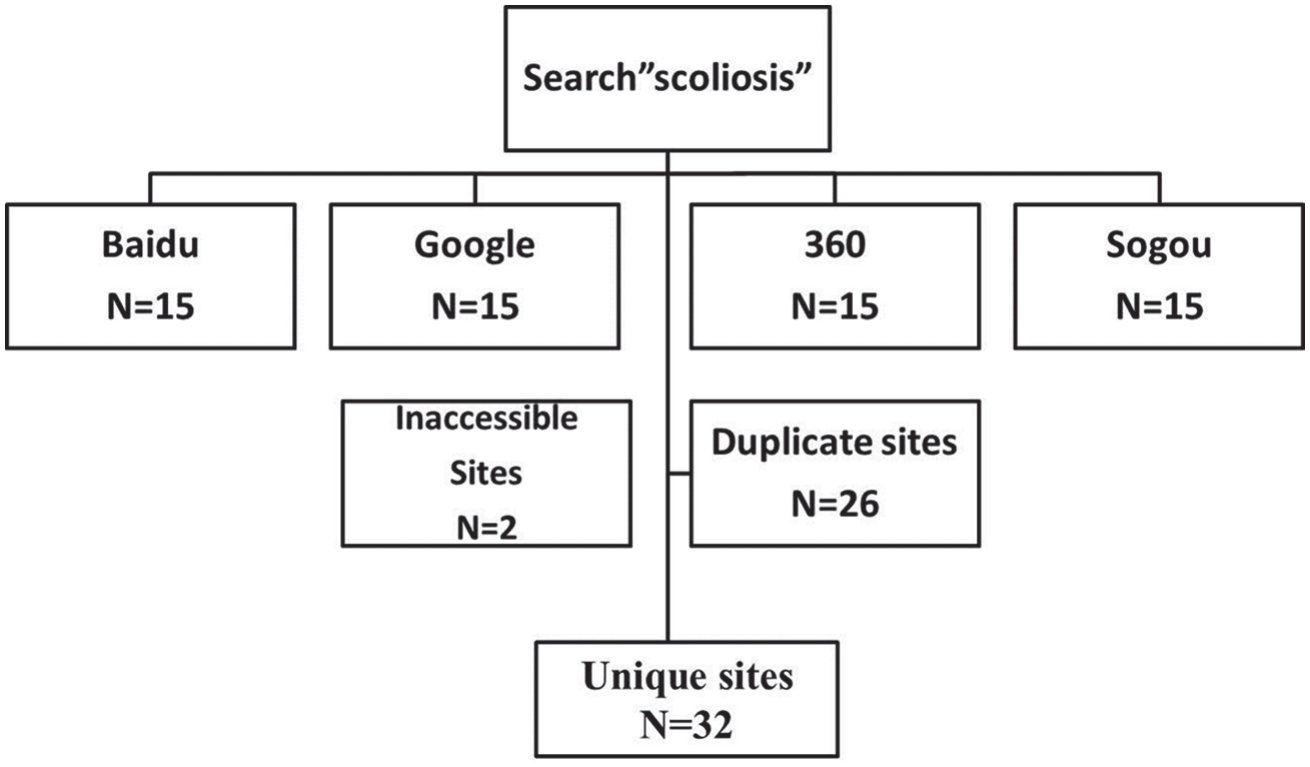
Flow chart of website search using the four most popular search engines.

The 4 most commonly used search engines in China were analyzed by a surgeon, these include Baidu (www.baidu.com), Google (www.google.com/hk), Sogou (www.sogou.com) and 360 (www.so.com). The term “scoliosis” in Chinese was searched in each search engine and the first 15 websites listed in Chinese were selected for further analysis (searches performed on January 11, 2013). Duplicate and inaccessible sites were excluded and finally a total of 32 websites were analyzed (Fig. 1). Following algorithms from Nason et al.[10], the websites were categorized as academic, physician, non-physician (allied health professionals, such as physiotherapist, occupational therapists, and alternative medical providers), commercial, media, discussion groups/social networks, and unspecified (ie. noncommercial websites that do not belong in the aforementioned categories established by independent individuals or organizations). Websites were assessed for accuracy and validity according to the DISCERN score and Journal of American Medical Association (JAMA) benchmark criteria according to the protocol provided in previous literature [14]. The DISCERN score is a short questionnaire with 16 questions, which enables patients and information providers to judge the quality of written information including the information on the Internet (S2 Questionnaire). For each question, 5 points were scored for the most positive answer and 1 point for the most negative answer. Four components were evaluated in the JAMA benchmark, including authorship, attribution, disclosure and currency. In addition, two experienced spine surgeons were asked to evaluate the quality of websites using the DISCERN instrument and JAMA benchmark.

### Statistics

Statistical analysis was performed using SPSS 13.0 (SPSS, Inc., Chicago, IL). Basic descriptive data are used to report results, such as proportions when reporting responses to questions regarding internet use. A binary logistic regression analysis was also constructed with “use of the Internet” as the dependent variable. With respect to the quality of online information, the summative scores calculated by two reviewers were tested for correlation with each other using Kendallτ. Mann-Whitney U tests and analysis of variance were used to determine statistical significance between the authorship categories (Non-physician, Physician, Commercial, Social media and Media groups). For all analyses, the level of significance used was p<0.05.

## Results

### Subjects

A total of 280 respondents (legal guardians of the child scoliosis patients) completed the survey with an average age of 36.7 years (range, 25–58 years). Sixty three respondents were between 20 and 30 years of age, 122 were between 30 and 40 years, 95 were over 40 years of age. Table 1 summarizes the demographic information gathered from the survey.

**Table 1:** Demographic details of the responses for the questionnaire.

		N	Percentage
Gender of patients	Female	242	86.43%
	Male	38	13.57%
Age distribution of respondents	20∼30 years	63	22.5%
	30∼40 years	122	43.6%
	Over 40 years	95	33.9%
Family location	Urban	184	65.7%
	Rural	96	34.3%
Highest level of guardian’s education	Primary school and below	41	14.6%
	Secondary school	109	38.9%
	High school	84	30.0%
	College and above	46	16.5%
Visit type	First visit	114	40.7%
	Follow-up	166	59.3%
Has your child had corrective surgery?	Yes	75	26.8%
	No	205	73.2%

### Analysis of questionnaire

Answers to questions related to the use of Internet were summarized in Table 2. The percentage of households with Internet access was 69.3% and that for patients with regular Internet use was 56.4%. In spite of that, 87.8% of those surveyed had used the Internet to search for information about scoliosis, and the percentage of searching prior to first clinical consultation was 65.7%. The respondents’ primary interest was the disease as a whole (63.9%).

**Table 2:** Results of assessment of the general Internet usage of the respondents.

		N	Percentage
Do you have internet access at home?	Yes	194	69.3%
	No	86	30.7%
Do you usually use the Internet?	Yes	158	56.4%
	No	122	43.6%
Have you searched the Internet for information on scoliosis?	Yes	246	87.8%
	No	34	12.2%
Had you searched prior to your first consultation?	Yes	184	65.7%
	No	96	27.5%
Who does the Internet search?	Myself	169	60.3%
	Through a relative or friend	77	31.3%
How did you find websites on scoliosis?	Search engine	198	70.7%
	Physicians	33	11.8%
	Other patients	15	5.3%
What do you search on the Internet?	Etiology	14	5%
	Prognosis	21	7.5%
	Treatment	32	11.4%
	General, not specific information on scoliosis	179	63.9%
Search engine used	Baidu	174	62.1%
	Google	35	12.5%
	Sogou	13	4.6%
	360	24	8.6%

Information regarding the respondents’ personal experience of using the Internet as a source of health information was summarized in Table 3. 68% of respondents considered the information useful. However, the average score of the websites analyzed was 41.47 (range: 10–78), indicating poor quality of information presented. In 23.9% of respondents, the online information caused variable degrees of anxiety. In addition, 71.1% of the respondents reported that the information available online generated more questions on the disease. Only 46.3% of the respondents shared the information with their physicians and very few of them (21.9%) would recommend the websites they have used.

**Table 3:** Results of questions assessing the search experience of respondents.

		N	Percentage
Was the Internet helpful?	Yes	169	68.7%
	No	77	31.3%
Did the Internet search create more anxiety?	Yes	59	23.9%
	No	187	76.1%
Did the search prompt more questions for this visit?	Yes	175	71.1%
	No	71	28.9%
Did you encounter confusing websites?	Yes	215	87.4%
	No	31	12.6%
Would you recommend sites after your search?	Yes	54	21.9%
	No	192	78.1%
Do you share the information with your doctor?	Yes	114	46.3%
	No	132	53.7%

Based on the binary logistic regression analysis, variables associated with Internet usage were college education, Internet access at home and urban residence (P<0.05) (Table 4). Other variables, including gender, age distribution of legal guardians, visit type, prior corrective surgery and regular Internet use were not associated with online information search (P>0.05).

**Table 4:** Binary logistic regression model for the use of the Internet as a source of scoliosis related information.

	Variables	No. of Internet use	Total no.	OR	P
Gender	Female	215	242	1.9	0.48
	Male	31	38	1	
Age distribution of respondents	20∼30 years	60	63	2.1	0.27
	30∼40 tears	115	122	1.8	0.36
	Over 40 years	71	95	1	
Education	Primary school and below	23	41	1	
	Secondary school	98	109	1.7	0.18
	High school	79	84	2.4	0.09
	College and above	46	46	3.8	0.041
Family location	Urban	174	184	3.4	0.027*
	Rural	72	96	1	
Visit type	First visit	95	114	0.8	0.67
	Follow up	151	166	1	
Prior corrective surgery	Yes	68	75	1	
	No	178	205	0.84	0.35
Internet access at home	Yes	191	194	4.5	0.013*
	No	55	86	1	
Regular Internet user	Yes	145	158	1.5	0.27
	No	101	122	1	

*: P<0.05

### Instrumentations for scoring of scoliosis-related websites

**Fig 2:**
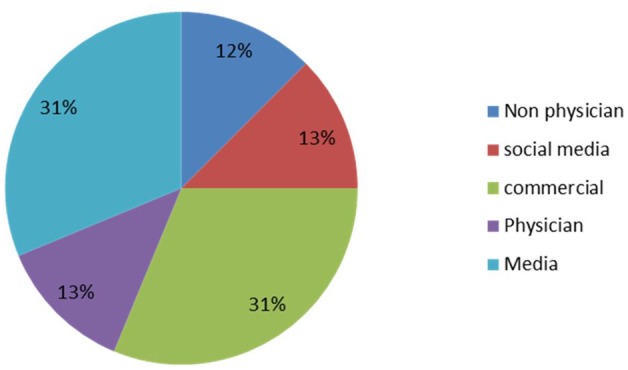
Description distribution of the websites evaluated.

A significant correlation was found between the two reviewers for both the DISCERN criteria and JAMA benchmark (τ = 0.813, P = 0.012 and τ = 0.635, P = 0.037, respectively). With respect to the types of authorship, commercial websites and media websites were the most common of the websites analyzed. Four of the websites were produced by physicians and 4 by non-physicians, while no academic website was identified (Fig. 2). The score for each authorship category was shown in Table 5. The mean score based on the DISCERN criteria was 27.9±11.7 (range: 16–55). Significant differences were observed between physician websites and media, non-physician and commercial websites. Social media websites showed satisfactory quality with an average DISCERN score of 43.3. Likewise, the average JAMA benchmark of physician websites was significantly higher than that of the other four groups (P<0.05).

**Table 5:** Average DISCERN score and JAMA benchmark in each description category.

Description category	Number	DISCERN score	JAMA benchmark
Non-physician	4	20.1	1.2
Physician	4	48.5*	3.8*
Commercial	10	25.2	1.6
Social media	4	43.2*	3.0*
Media	10	19.4	1.8

*: P<0.05

## Discussion

As technology revolutionized our lives, the Internet has become the most popular source of information, including health-related information [4]. The Internet is a convenient source of information for disease management and a potential health education tool. Patients’ Internet access may change the patient-physician relationship, such as patients’ resistance to physician advice and impeding the shared decision-making process between physicians and self-educated patients. Nason et al.[10] reported a 20-fold increase in the number of scoliosis related websites in 8 years compared to that reported in 2005 [4]. However, the massive amount of information available to patients also leads to increasing difficulty in selecting for the accurate information. Mathur *et al*.[4] assessed the quality of information available on the Internet regarding scoliosis and concluded that it was mostly of poor quality. Nason et al.[10] also noticed that the overall quality of information regarding scoliosis remained poor despite an exponential increase in the number of sites available. In addition, characteristics of patients using Internet should also be clarified, thus healthcare information providers will be able to communicate with patients more effectively.

This is the first study focusing on how scoliosis patients in China used the Internet to search for scoliosis related information and the quality of such online information. There have been various other reports on the prevalence of using the Internet as a source for other medical information in China. Gao et al.[15] reported that 88.7% of pregnant women in China retrieved information about pregnancy while Liu et al.[16] showed that 36.9% of Chinese epilepsy patients searched for epilepsy related medical information. In this study, the prevalence of Internet use in Chinese scoliosis patients was 87.8%, which is much higher than the 58% prevalence previously described in an Irish population [8]. The percentage of patients who searched for related medical information online prior to their first outpatient consultation was as high as 65.7%. Even though scoliosis can be easily diagnosed in community hospitals, the orthopedic doctors in these smaller hospitals may not be able to give patients enough information on the disease, and therefore, patients are likely to demand more detailed information on the disease. A previous study found that older patients were less likely to use the Internet as a source of health information, the respondents in this study were younger (66.1% younger than 40 years) and are more competent in using the Internet [11]. In addition, our hospital is located in one of the most developed regions in China, and therefore, our patients may have better economic situation and more convenient access to the Internet. Moreover, patients admitted to our hospital are often referred to our hospital by Internet search, and thus the patient population within the present study is more likely to be frequent Internet users.

In this study, urban residence, Internet access at home and college education were considered to be potential predictors for Internet use according to multivariable analysis. The former two of these factors were also confirmed by binary logistic regression. China is a developing country with an imbalanced economic development, which leads to drastic differences in economic infrastructure, the modality of healthcare access and Internet usage. Wang et al.[17] and Cheung et al.[18] both revealed differences in patients’ response between urban and rural areas when evaluating spinal questionnaires in China. Family location in either a urban or rural setting may also be related to Internet accessibility. The China Internet Network Information Center reported in 2013 that rural Internet users only accounted for 27.6% of total users, despite the fact that 51.3% of the Chinese population reside in a rural setting (2010 census), supporting the hypothesis that urban residence is a major factor contributing to the likelihood of online search for scoliosis [19]. However, it was noted in our results that use of Internet to search for scoliosis related information was not necessarily influenced by the frequency of Internet use, implying that the demand for health related information provides motivation for some patients to use the Internet.

The quality of online information on scoliosis in China was also evaluated in this study, demonstrating poor quality in terms of accuracy. No academic sources were found within the first 15 websites listed on each search engine and the average DISCERN score for all websites analyzed was 27.6, which is significantly lower than that reported in the United Kingdom and Ireland [9,10]. The average DISCERN score reported by Wellburn et al.[9] was 37.57, larger than that reported in our study since the websites collected in his study were recommended by consultants. These results highlighted the dilemma that patients in China have to encounter when they are in need of more scoliosis related information. Similar to our conclusions, Welburn et al.[9] also reported that the websites recommended by UK NHS Consultants were of poor quality in terms of scoliosis related information, especially the information related to conservative management methods for scoliosis, such as bracing, which has been demonstrated to effectively control the progression of scoliosis based on recent Cochrane reviews [20]. One possible explanation for the poor quality of online information is the lack of regulation of Internet based information. For instance, a health related website can be easily set up without supervision from authorities such as the Ministry of Health. In the present study, 43% of the websites was commercially driven or set up by non-physicians. These websites, along with some media websites, make profits through advertising. Some of these advertisements provide links to unprofessional treatment options. As demonstrated by Wellburn et al.[9] and Nason et al.[10], websites displaying a Health on Net Foundation code (HONcode), a website certification evaluated by medical experts, scored more highly than websites without the code, indicating that the information provided on websites without HONcode may not be evidence based, accurate, or trustworthy. However, there is no such certification system of websites similar to HONcode which could be used to distinguish the quality of websites.

In our survey, 87.4% of patients who searched the Internet encountered confusing websites. The average score for the usefulness of the Internet was 41.47, which demonstrated the poor quality of scoliosis related website in China. The results of this study are consistent with various surveys and observational studies across different cultures and for different diseases [2–4,7,10,16,21]. Mathur et al.[4], Wellburn et al. [9]and Nason et al.[10] all demonstrated limited quality and poor value of information regarding scoliosis in the United States, United Kingdom and Ireland. Liu et al.[16] implied that most websites associated with epilepsy in China did not comply with accepted standards and may contain inaccurate information. All of these studies made apparent the challenge faced by medical professionals when consulting with patients who have been misled by inaccurate online information. One solution to this problem is for the doctors to recommend good quality online information to patients that will help guide patients through a shared decision-making process, as is the case for UK NHS consultants for AIS patients [9].

Less than half of the respondents (46.3%) discussed the information they found online with their medical provider. This is a slightly higher proportion than that found in various other surveys (9 to 41%) [22,23]. There is potentially a trend that the practice of discussing Internet information with one’s physician has become more acceptable within recent years.

To the best of our knowledge, this is the first study focusing on scoliosis-related online information in China, a typical developing country. Despite that Wellbure et al. [9] have studied the websites recommended by UK NHS consultants, the websites analyzed in this study were obtained using online search engines. In addition, the self-reported instruments were also used to study the characteristics of patients using Internet prior to consulting. Therefore the quality of websites could be better evaluated from both the perspective of the patients and consultants.

The limitations of the present study should also be mentioned. This study was conducted using a small sample of patients from a national spinal center in China, and may not be representative of the general population in China. Moreover, as aforementioned, our patients may have better economic situation and more convenient access to the Internet since our center located in a more developed area; patients admitted to our hospital are often referred to our hospital by Internet search, leading to a potential bias. A further multi-center survey on Internet use in China should be performed. Responses were self-reported and the validity and reproducibility of the survey were not verified. In addition, a further study was needed to stress the influence of online information by comparing the physician’s experience with consulting patients who read websites produced by physicians with those who read health information from a commercial or social media site.

## Conclusions

In conclusion, the results of this study showed that 87.8% of the scoliosis patients in our outpatient clinics have searched for scoliosis related information on the Internet. Urban patients, higher education and Internet access at home were identified as potentials predictors for using the Internet as a health information tool. However, the overall quality of online scoliosis related information was poor and confusing. Physician based websites may contain more reliable information compared to commercial and non-physician based websites. It may be beneficial for both patients and healthcare providers if governments, hospitals, physicians, and Internet service providers collaborated to create convenient, reliable Internet platforms for patients.

## Supporting Information

S1 Questionnaire Questionnaires distributed to outpatients in this study. (PDF)

S2 Questionnaire DISCERN scoring system used in this study. (PDF)
