# Supplementary material for: Scoliosis Related Information on the Internet in China: Can Patients Benefit from This Information?
Source: PLoS One. 2015 Feb 17;10(2):e0118289. doi: 10.1371/journal.pone.0118289 (PMC4331504; doi:10.1371/journal.pone.0118289)
Supplement: S1 Questionnaire — (PDF) [file pone.0118289.s001.pdf]

脊柱侧弯患者网络使用情况调查

为了了解您对脊柱侧弯相关知识的获取渠道，我们邀请您填写一份调查问卷，希望可以通过这份问卷提高网络上脊柱侧弯相关知识的质量，谢谢您！

您的孩子的性别：☐ 男　　☐ 女　　年龄（周岁）：

你的性别：☐ 男　　☐ 女

你的年龄：☐20-30 岁　　☐30-40 岁　　☐40 岁以上

1、 您目前的学历是什么？
☐小学　　☐初中　　☐高中　　☐大学及以上

2、 您的孩子这次来看病属于哪种情况？
☐第一次发现脊柱侧弯
☐以前发现脊柱侧弯，本次是随访
☐已经进行脊柱侧弯手术，是术后复查

3、 如果您的孩子已经进行了脊柱侧弯手术，术后有没有并发症？
☐有　　☐没有

4、 您的家庭住址属于：
☐城市　　☐农村

5、 您的家庭中是否有网络覆盖？
☐有　　☐没有

6、 您是否经常使用网络？
☐是　　☐不是

7、 你之前是否在网络上搜索过脊柱侧弯相关的知识？
☐有　　☐没有

8、 如果您曾经搜索过侧弯相关知识，这样的搜索是谁进行的？
☐我自己　　☐朋友或亲戚的帮助

9、 你在刚刚发现孩子脊柱侧弯，但没有看门诊时，是否上网搜索相关知识？
☐有　　☐没有

10、 你是否与您的主诊医生共享你查到的信息？
☐有　　☐没有

11、 如果您使用过网络获取脊柱侧弯相关知识，你觉得有没有给你带来更多的疑问？

☐有 ☐没有

12、 您是怎样找到脊柱侧弯相关网站的？

☐网络搜索引擎

☐朋友推荐

☐医生推荐

☐病友推荐

13、 您经常使用什么搜索引擎搜索侧弯相关知识？

☐百度 ☐谷歌 ☐搜狗 ☐360 搜索

14、 您觉得上网络搜索脊柱侧弯知识是否有帮助？

☐有 ☐没有

15、 您觉得网络上的知识是否会让您对孩子的病情更焦虑？

☐会 ☐不会

16、 您是否给病友推荐过比较好的脊柱侧弯网站？

☐有 ☐没有

17、 您是否曾经遇到令人困惑的网站？

☐有 ☐没有

18、 你上网搜索脊柱侧弯相关知识，主要是为了什么？

☐疾病的病因

☐侧弯的预后如何

☐侧弯的治疗手段

☐希望了解比较全面的信息

19、 您觉得网上的知识在多大程度上可以解答您的问题？请在下方的直线上标出来，最左边表示完全不能解答，最右边表示可以完全解答。

0%，完全不能解答 100%，可以完全解答

感谢您参与我们的调查，您的参与是对我们最大的支持！

我们一贯致力于为患者提供最好的脊柱侧弯诊疗方案，感谢您为这个目标做出贡献！
