# Supplementary material for: Scoliosis Related Information on the Internet in China: Can Patients Benefit from This Information?
Source: PLoS One. 2015 Feb 17;10(2):e0118289. doi: 10.1371/journal.pone.0118289 (PMC4331504; doi:10.1371/journal.pone.0118289)
Supplement: S2 Questionnaire — (PDF) [file pone.0118289.s002.pdf]

DISCERN

An instrument for judging the quality of written consumer health information on treatment choices

Funded by The British Library

For further information please contact:

Sasha Shepperd

University of Oxford

Division of Public Health and Primary Health Care

Institute of Health Sciences

Old Road

Headington

Oxford OX3 7LF

Section 1

IS THE PUBLICATION RELIABLE?

1 Are the aims clear?

No		Partially		Yes
1	2	3	4	5

HINT Look for a clear indication at the beginning of the publication of:

- what it is about
- what it is meant to cover (and what topics are meant to be excluded)
- who might find it useful.

If the answer to Question 1 is 'No', go directly to Question 3

2 Does it achieve its aims?

No		Partially		Yes
1	2	3	4	5

HINT Consider whether the publication provides the information it aimed to as outlined in Question 1.

3 Is it relevant?

No		Partially		Yes
1	2	3	4	5

HINT Consider whether:

- the publication addresses the questions that readers might ask
- recommendations and suggestions concerning treatment choices are realistic or appropriate.

4 Is it clear what sources of information were used to compile the publication (other than the author or producer)?

No		Partially		Yes
1	2	3	4	5

HINT

- Check whether the main claims or statements made about treatment choices are accompanied by a reference to the sources used as evidence, e.g. a research study or expert opinion.
- Look for a means of checking the sources used such as a bibliography/reference list or the addresses of the experts or organisations quoted.

Rating note: In order to score a full '5' the publication should fulfil both hints. Lists of *additional* sources of support and information (Q7) are not necessarily sources of *evidence* for the current publication.

5 Is it clear when the information used or reported in the publication was produced?

No		Partially		Yes
1	2	3	4	5

HINT Look for:

- dates of the main sources of information used to compile the publication
- date of any revisions of the publication (but not dates of reprinting)
- date of publication (copyright date).

Rating note: The hints are placed in order of importance – in order to score a full '5' the dates relating to the first hint should be found.

6 Is it balanced and unbiased?

No		Partially		Yes
1	2	3	4	5

HINT Look for:

- a clear indication of whether the publication is written from a personal or objective point of view
- evidence that a *range* of sources of information was used to compile the publication, e.g. more than one research study or expert
- evidence of an external assessment of the publication.

Be wary if:

- the publication focuses on the advantages or disadvantages of one particular treatment choice without reference to other possible choices
- the publication relies primarily on evidence from single cases (which may not be typical of people with this condition or of responses to a particular treatment)
- the information is presented in a sensational, emotive or alarmist way.

7 Does it provide details of additional sources of support and information?

No		Partially		Yes
1	2	3	4	5

HINT Look for suggestions for further reading or for details of other organisations providing advice and information about the condition and treatment choices.

8 Does it refer to areas of uncertainty?

No		Partially		Yes
1	2	3	4	5

HINT

- Look for discussion of the gaps in knowledge or differences in expert opinion concerning treatment choices.
- Be wary if the publication implies that a treatment choice affects everyone in the same way, e.g. 100% success rate with a particular treatment.

Section 2

HOW GOOD IS THE QUALITY OF INFORMATION ON TREATMENT CHOICES?

N.B. The questions apply to the treatment (or treatments) described *in the publication.* Self-care is considered a form of treatment throughout this section.

9 Does it describe how each treatment works?

No		Partially		Yes
1	2	3	4	5

HINT Look for a description of how a treatment acts on the body to achieve its effect.

10 Does it describe the benefits of each treatment?

No		Partially		Yes
1	2	3	4	5

HINT Benefits can include controlling or getting rid of symptoms, preventing recurrence of the condition and eliminating the condition, both short-term and long-term.

11 Does it describe the risks of each treatment?

No		Partially		Yes
1	2	3	4	5

HINT Risks can include side-effects, complications and adverse reactions to treatment, both short-term and long-term.

12 Does it describe what would happen if no treatment is used?

No		Partially		Yes
1	2	3	4	5

HINT Look for a description of the risks and benefits of postponing treatment, of watchful waiting (i.e. monitoring how the condition progresses without treatment) or of permanently forgoing treatment.

13 Does it describe how the treatment choices affect overall quality of life?

No		Partially		Yes
1	2	3	4	5

HINT Look for:

- description of the effects of the treatment choices on day-to-day activity
- description of the effects of the treatment choices on relationships with family, friends and carers.

14 Is it clear that there may be more than one possible treatment choice?

No		Partially		Yes
1	2	3	4	5

HINT Look for:

- a description of who is most likely to benefit from each treatment choice mentioned, and under what circumstances
- suggestions of alternatives to consider or investigate further (including choices not fully described in the publication) before deciding whether to select or reject a particular treatment choice.

15 Does it provide support for shared decision-making?

No		Partially		Yes
1	2	3	4	5

HINT Look for suggestions of things to discuss with family, friends, doctors or other health professionals concerning treatment choices.

Section 3

OVERALL RATING OF THE PUBLICATION

16 Based on the answers to all of the above questions, rate the overall quality of the publication as a source of information about treatment choices

Low		Moderate		High
Serious or extensive shortcomings		*Potentially important but not serious shortcomings*		*Minimal shortcomings*
1	2	3	4	5

Copyright British Library and the University of Oxford 1997
